# Dynamic Characteristics of Mechanical Ventilation System of Double Lungs with Bi-Level Positive Airway Pressure Model

**DOI:** 10.1155/2016/9234537

**Published:** 2016-08-29

**Authors:** Dongkai Shen, Qian Zhang, Yan Shi

**Affiliations:** ^1^School of Automation Science and Electrical Engineering, Beihang University, Beijing 100191, China; ^2^Beijing Engineering Research Center of Diagnosis and Treatment of Respiratory and Critical Care Medicine, Beijing Chaoyang Hospital, Beijing 100043, China

## Abstract

In recent studies on the dynamic characteristics of ventilation system, it was considered that human had only one lung, and the coupling effect of double lungs on the air flow can not be illustrated, which has been in regard to be vital to life support of patients. In this article, to illustrate coupling effect of double lungs on flow dynamics of mechanical ventilation system, a mathematical model of a mechanical ventilation system, which consists of double lungs and a bi-level positive airway pressure (BIPAP) controlled ventilator, was proposed. To verify the mathematical model, a prototype of BIPAP system with a double-lung simulators and a BIPAP ventilator was set up for experimental study. Lastly, the study on the influences of key parameters of BIPAP system on dynamic characteristics was carried out. The study can be referred to in the development of research on BIPAP ventilation treatment and real respiratory diagnostics.

## 1. Introduction

Mechanical ventilation is an intervention used to help patients breathe in ICU. The aim of mechanical ventilation is to improve gas exchange rate of patients' respiratory system and help critically ill patients or patients with various forms of respiratory disorders reduce breathing work [1, 2]. Although many years have been spent since mechanical ventilation was used in ICU for the first time, it is always difficult to choose the best ventilation mode (or combination of modes) for patients and to adjust the settings of ventilator as the conditions ‎[1]. But the development of new ventilation techniques has been never stopped.

At present, there are a variety of mechanical ventilations controlled models, such as Pressure Controlled Ventilation (PCV) and Volume Controlled Ventilation (VCV) ‎[3]. On the base of PCV, the models of Pressure Support Ventilation (PSV), Pressure Released Ventilation (PRV), Pressure Regulated Volume Ventilation (PRVC) and Bi-Level Positive Airway Pressure (BIPAP) Controlled Ventilation are developed [4–10]. Among them, as a similar universal breathing model ‎[11], BIPAP gets a wide range of application due to the characteristics of pressure control, well man-machine coordination [12–17].

In recent researches on mechanical ventilation systems, it was considered that human had only one lung for easy analysis [18–20]. However, the respiratory organ of human consists of right lung and left lung, and respiratory parameters of one lung are different from another lung's parameters. Furthermore, in breathing process, respiratory dynamics of one lung are affected by another lung.

As is known, the coupling effects of double lungs on dynamic characteristics of mechanical ventilation system are of significant for the security and effect of mechanical ventilation, which can not be illustrated when just only one lung is considered [21, 22].

Furthermore, the models of the mechanical ventilation systems with just only one lung are not comprehensive or precise which reduces their applicability and versatility and makes great error on the respiratory parameter identification [23–26].

However, till now, there are few studies on the mechanical ventilation system with double lungs.

In this paper, to lay a theoretical foundation for safe, efficient mechanical ventilation and accurate respiratory parameter identification, firstly, a mathematical model of a mechanical ventilation system, which consists of double lungs and a bi-level positive airway pressure (BIPAP) controlled ventilator, was proposed. To verify the mathematical model, a mechanical ventilation system prototype was set up for experimental study. Lastly, the influence of parameters of ventilation system on dynamic characteristics was discussed.

## 2. Introduction and Modeling of Double-Lungs Mechanical Ventilation System

### 2.1. Introduction of Mechanical Ventilation System of Double Lungs

**Figure 1:**
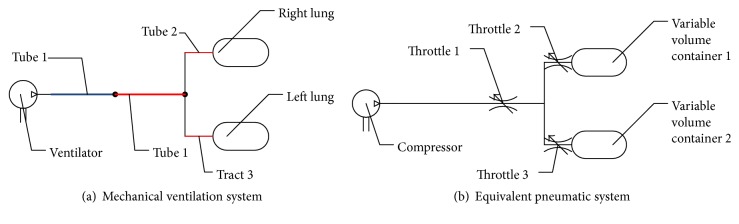
Structures of mechanical ventilation system with double lungs and equivalent pneumatic system.

A BIPAP ventilation system of double lungs, as shown in Figure 1(a), is composed of two human lungs, a ventilator, three flexible tubes, and a respiratory tract. In inspiration process, mechanical ventilation with positive pressure made by the ventilator forces air flow into the human lungs. And then, in expiration process, air is expelled through an exhalation valve to the atmosphere due to high pressure of double lung.

According to these functions, an air compressor is used to imitate ventilator, three throttles are used to imitate exhalation valve and two tubes, and two lungs can be regarded as two variable volume containers. The matching of settings of ventilator to actual parameters of the respiratory system has an effect on efficiency of ventilation which is mainly made up of respiratory resistance (*R*_*r*_) and compliance (*C*) ‎[27] as described above.

However, the respiratory resistance (*R*_*r*_), which varies with time, can be replaced as friction loss which happens in the respiratory tube ‎[28]. So, each respiratory tube can be regarded as two equivalent throttles which connects with each lung and the ventilator. The respiratory compliance (*C*) of the tube and respiratory tract, which also varies with time, can be neglected [29–31], because the pressure in each lung of ventilation system is about 2 cmH_2_O to 40 cmH_2_O. Therefore, a mechanical ventilation system with two lungs can be simplified to an equivalent pneumatic system [18–26, 32], as shown in Figure 1(b).

### 2.2. Mathematical Modeling of Mechanical Ventilation System with Double Lungs

In order to facilitate research, the study was conducted under the assumptions that no air leaks during working process, and the dynamic process is a quasi-balanced process ‎[33].

#### 2.2.1. Flow Equation

The air mass flow can be obtained by flow equation of throttles after air flows through the LAVAL nozzle. For each lung, when *pd*/*pu* > *b*, air flow is subsonic and when *pd*/*pu* ⩽ *b*, air flow is sonic ‎[28]. According to the calculation result, *pd*/*pu* is bigger than *b* invariably. The air mass flow in total (*q*_*s*_) is the air mass flow which flows into the right lung adds up the left lung. Therefore, mass flow equations of the system can be reached by

(1) qr=ArePru1T·2kk−1·1RPrdPru2/k−PrdPruk+1/k,ql=AlePlu1T·2kk−1·1RPldPlu2/k−PldPluk+1/k,qs=qr+ql.

#### 2.2.2. Pressure Equation

The research is under the assumption that the inspiration and respiration are isothermal processes. The differential expression of the Clapeyron equation (*PV* = *mRθ*) is given by ‎[28]

(2) $$\begin{array}{c} \frac{dp}{dt}=\frac{1}{V}R\theta q-\frac{mR\theta }{V^{2}}\frac{dV}{dt}. \end{array}$$

We can transform (2); then the pressure in right lung and left lung can be given by

(3) dprdt=RθqVrVr2+CrmrRθ,dpldt=RθqVlVl2+ClmlRθ.

#### 2.2.3. Volume Equation

According to the definition of the respiratory compliance (*C*), the respiratory compliance (*C*) can be described as ‎[34]

(4) $$\begin{array}{c} C=\frac{dV}{dp}. \end{array}$$

Then the volume of right lung and left lung can be calculated by

(5) dVr=Crdpr,dVl=Cldpl.

## 3. Verification of Model with Double Lungs

### 3.1. Experimental Apparatus and Study

**Figure 2:**
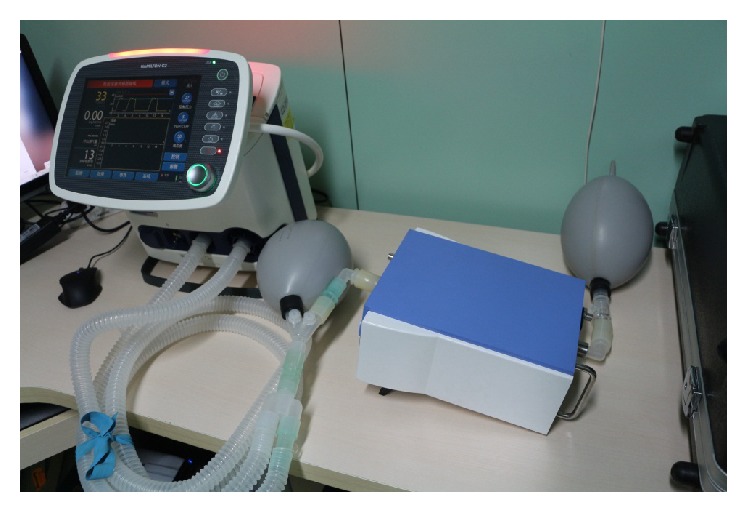
Experiment apparatus.

To verify the mathematical model described above, an experiment for the ventilation has been conducted. As shown in Figure 2, the experimental apparatus consists of a BIPAP ventilator, two tubes, a pressure-flow sensor, two lung simulators, and a computer.

The pressure-flow sensor called FlowAnalyser ventilator tester PF-300 is provided by the* imtmedical* company. It is used to detect flow, pressure, temperature, humidity, and O_2_ concentrations bidirectionally. The ventilator is provided by* HAMILTON*. It is a universal ventilation solution for all patient groups and offers appropriate tidal volumes as low as 2 mL with the aim of reducing lung damage.

There are several main settings of ventilator: inspiratory positive airway pressure (IPAP), expiratory positive airway pressure (EPAP), breaths per minute (BPM), inspiratory time (*T*_*i*_), and rise time of pressure (*T*_*r*_), and they have been set already before the experiment. IPAP are set to 22 cmH_2_O. EPAP are set to 4 cmH_2_O. BPM are set to 20 per minute. And the inspiration time and the pressure rise time are set to 1 s and 0.3 s, respectively. When the conditions of ventilation system get stable, data acquisition and preservation are executed.

### 3.2. Simulation of the Ventilation System

**Figure 3:**
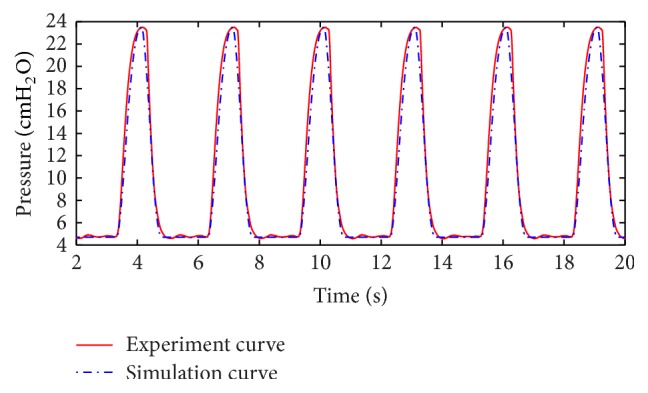
Curve and fitted curve of air pressure in one lung simulator.

**Figure 4:**
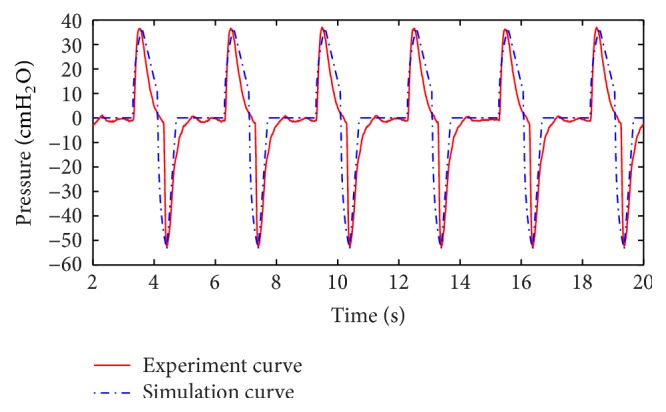
Curve of air mass flow of one lung simulator.

According to the mathematical model and the experiment described above, the curve of pressure in one lung from experiment and the fitted curve of the air pressure in one lung simulator from simulation are shown in Figure 3. Also the curve of air mass flow in one lung simulator from experiment and simulation is shown in Figure 4.

In addition, the two lung simulators are the same. In other words, for each lung the respiratory compliance (*C*) and the inlet diameter (*d*) are the same. And the initial values of the parameter in simulation are the same as those in experiment.

The software, MATLAB/Simulink, is used for simulation.

### 3.3. Analysis

From Figures 3 and 4, the following can be summarized.

1. The simulation results are in accordance with the experimental results. It verifies the mathematical model can be used in this research.
2. As IPAP and EPAP offered by the ventilator are 23.5 cmH_2_O and 4.5 cmH_2_O, there exists little error, but the error can be neglected, and therefore the experiment results are authentic and reliable.
3. The reason of difference between experimental results and simulation results is that there is a little air leakage during the working process in the experiment.

## 4. Influence on Dynamic Characteristics

As humans have two lungs, different parameters between two lungs affect dynamic characteristics of the system. So it is necessary to study the effects of several main parameters on the dynamics of the ventilation system of the two different lungs in different conditions.

Since there are so many articles and studies about dynamic characteristics of ventilation system of one lung at present, it does not make any sense to study these dynamic characteristics of each lung simulator in this ventilation system. Among all kinds of dynamic characteristics, the ratio of air mass flow in right lung simulator (*q*_*r*_) to air mass flow in total (*q*_*s*_) is focused on in this study, as well as the ratio of tidal volume of right lung simulator (*V*_Tr⁡_) to tidal volume of the sum of two lung simulators (*V*_Ts_).

### 4.1. Influence of the Key Parameters of Lung Simulators on Dynamic Characteristics

**Figure 5:**
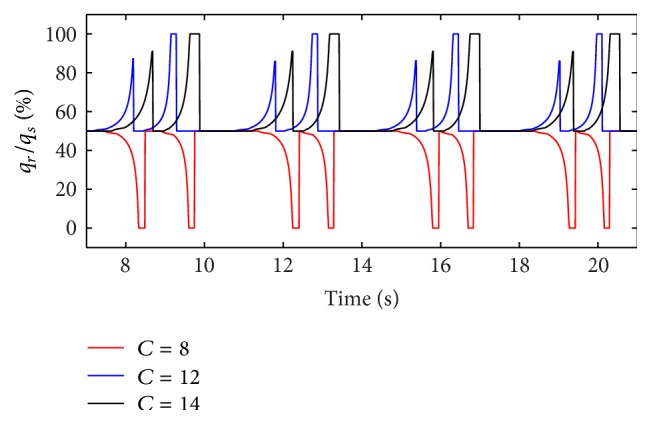
Influence of *C* on air mass flow ratio.

In this study, the respiratory compliance (*C*) and the inlet diameter (*d*) of one throttle would be changed, and the other lung simulator remains the same, which would be 10 mL/cmH_2_O and 3.2 mm, respectively. In addition, the values of the key parameters like EPAP, IPAP, BPM, *T*_*i*_, and *T*_*r*_ must be kept constant which is described as above.

(*1) Influence of the Respiratory Compliance of One Lung Simulator on Dynamic Characteristics*. Firstly, the inlet diameter (*d*) of two throttles is set to 3.2 mm. When the respiratory compliance is set to 8 mL/cmH_2_O, 12 mL/H_2_O, and 14 mL/cmH_2_O, the simulation results of the ratio of the air mass flow in right lung simulator (*q*_*r*_) to the air mass flow in total (*q*_*s*_) are illustrated in Figure 5.

**Figure 6:**
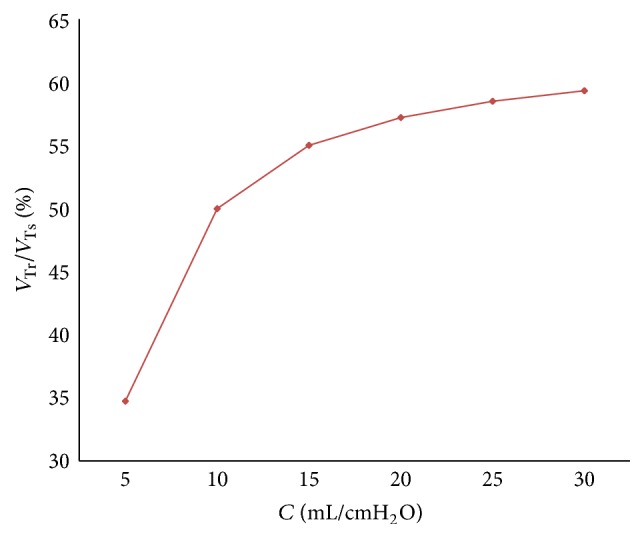
Influence of *C* on tidal volume ratio of different *C*.

The relation between respiratory compliance and the ratio of tidal volume of right lung simulator (*V*_Tr⁡_) to tidal volume of the sum of two lung simulators (*V*_Ts_) is studied, with the results shown in Figure 6, when the value of compliance (*C*) is set to 5 mL/cmH_2_O–30 mL/cmH_2_O, every 5 mL/cmH_2_O.

From Figures 5 and 6, the flowing can be seen.

As the ventilation system has inspiration process and expiration process, the ratio of air mass flow has two fluctuations in one period. When respiratory compliance (*C*) of right lung simulator is bigger than the left lung simulator, that is to say, respiratory compliance (*C*) of right lung simulator is bigger than 10 mL/cmH_2_O, the ratio of air mass flow would be 50% to 100%. The air mass flow in right lung simulator is more than air mass flow in left lung simulator. Otherwise, the ratio would be smaller than 50%, and the air mass flow in right lung simulator would be less than the left simulator. If the respiratory compliance (*C*) of right lung simulator is the same with left lung simulator, the ratio would keep 50% unchanged.

Furthermore, when the air mass flow of one lung simulator comes to be 0, the other lung simulator still has air mass flow. That is the reason why the ratio can be 0 and 100%.

**Figure 7:**
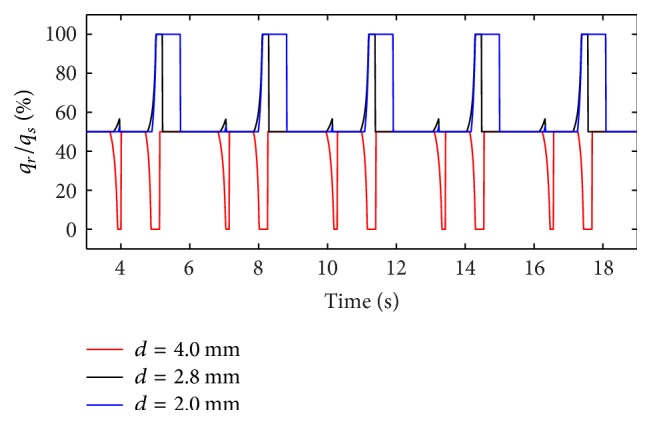
Influence of *d* on air mass flow ratio.

Finally, with a rise of respiratory compliance (*C*) of right lung simulator, the ratio of tidal volume increases. When respiratory compliance (*C*) of right lung simulator is smaller than left lung simulator, the ratio rises sharply. When respiratory compliance (*C*) of right lung simulator catches up with the left lung simulator, the growth of the ratio has been moderated slowly.

(*2) Influence of Diameter of Inlets of Two Lungs on Dynamic Characteristics*. As described above, the air mass flow is mainly influenced by the diameter (*d*) of inlets of two lungs. The diameter (*d*) of inlet of right lung is set to 2.0 mm, 2.8 mm, and 4.0 mm. The simulation results of the ratio of the air mass flow in right lung simulator (*q*_*r*_) to the air mass flow in total (*q*_*s*_) are shown in Figure 7.

**Figure 8:**
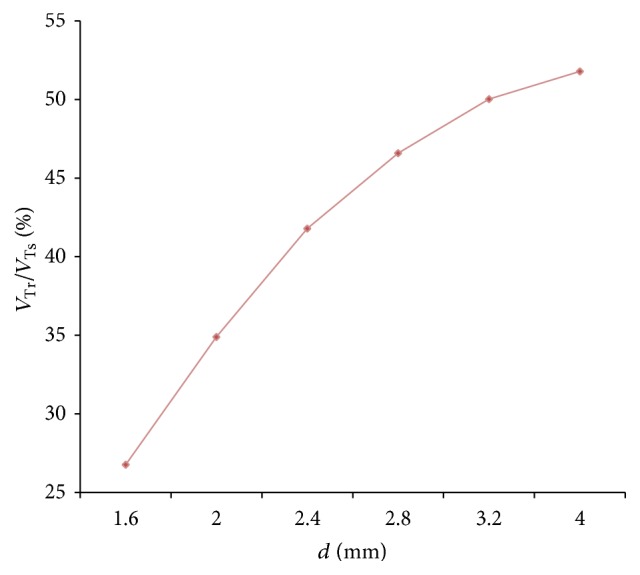
Influence of *d* on tidal volume ratio of different *d*.

The relation between respiratory compliance and the ratio of tidal volume of right lung simulator (*V*_*r*_) to tidal volume of the sum of two lung simulators (*V*_*s*_) is also studied, when *d* is set to 1.6 mm–4 mm, as illustrated in Figure 8.

As shown in Figures 7 and 8, the following is obvious.

Firstly, there are two processes in the ventilation system, so the ratio of air mass flow has two fluctuations in one period. When the diameter (*d*) of the effective area of the equivalent right throttle is bigger than the left throttle, the ratio of air mass flow would be 0% to 50%. On the contrary, if the diameter (*d*) of the effective area of the equivalent right throttle is smaller than the left throttle, the ratio would be 50% to 100%. When the diameter (*d*) of the effective area of the equivalent right throttle is the same as left throttle, the ratio would remain 50%.

Furthermore, the same as the influence of respiratory compliance (*C*), when the air mass flow of one lung simulator comes to be 0, the other lung simulator still has air mass flow. So the curve of the ratio of air mass flow can reach 0 and 100%. The larger gap between the diameters (*d*) of the effective area of the two equivalent throttles is the longer time of the ratio to be 0 or 100% that would stay.

Lastly, as a rise of the diameter (*d*) of the effective area of the equivalent right throttle, the ratio of tidal volume increases.

### 4.2. Influence of the Key Parameters of Ventilator on Dynamic Characteristics

So far, the dynamic characteristics which have been studied are under the fixed condition of ventilator. However, the key parameters of ventilator can make a difference on dynamic characteristics of mechanical ventilation system of two different lung simulators. Therefore, it is of great necessity to study the influence of the key parameters of ventilator on two different lung simulators.

#### 4.2.1. Influence of IPAP on Dynamic Characteristics

**Figure 9:**
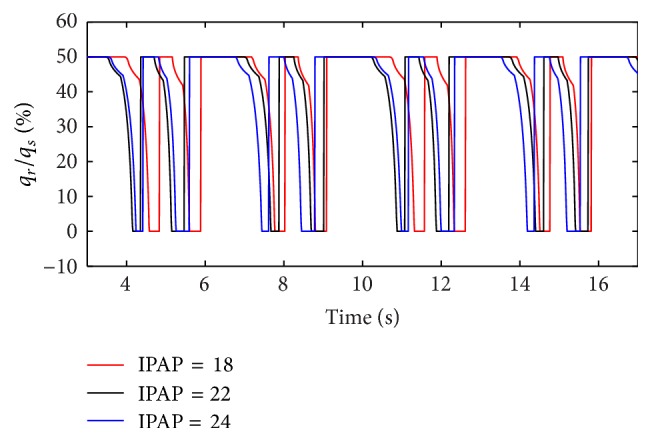
Influence of IPAP on air mass flow when *C* is set to 5 mL/cmH_2_O.

**Figure 10:**
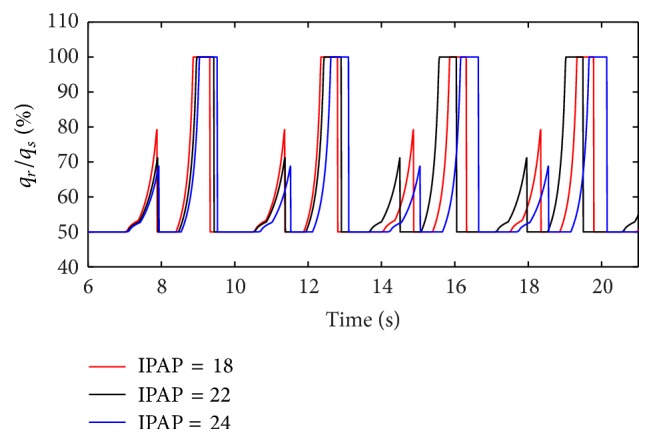
Influence of IPAP on air mass flow when *C* is set to 20 mL/cmH_2_O.

The IPAP is set to 18 cmH_2_O, 22 cmH_2_O, and 24 cmH_2_O. The respiratory compliance (*C*) of left lung simulator keeps to 10 mL/cmH_2_O, and the diameters (*d*) of the effective area of the equivalent left throttle keep to 3.2 mm.

(*1) Influence of IPAP under Different Respiratory Compliance (C) on Dynamic Characteristics*. When the respiratory compliance (*C*) of right lung simulator is set to 5 mL/cmH_2_O and 20 mL/H_2_O, the result of the ratio of air mass flow is shown as in Figures 9 and 10.

**Figure 11:**
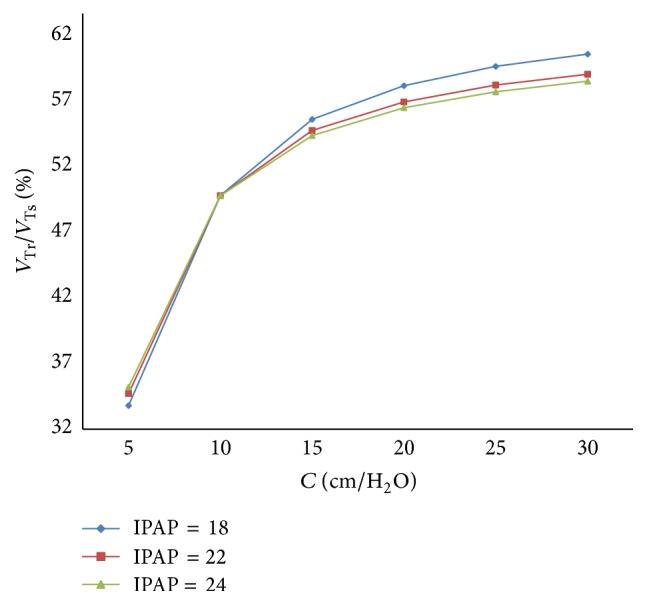
Influence of IPAP on tidal volume of different *C*.

With an increase in the respiratory compliance (*C*) of right lung simulator, the results of the ratio of tidal volume under different IPAP are shown in Figure 11.

**Figure 12:**
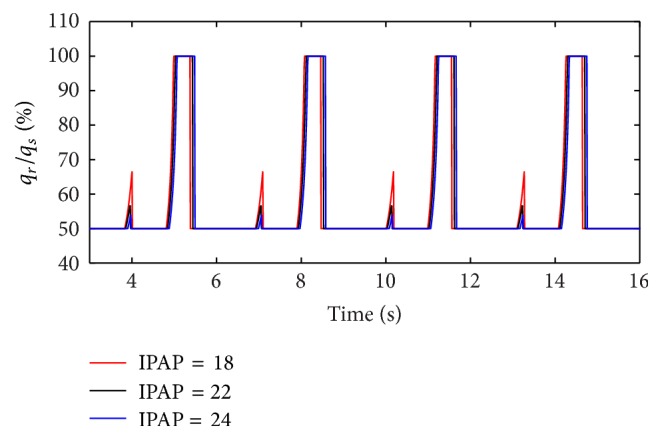
Influence of IPAP on air mass flow when *d* is set to 2.4 mm.

**Figure 13:**
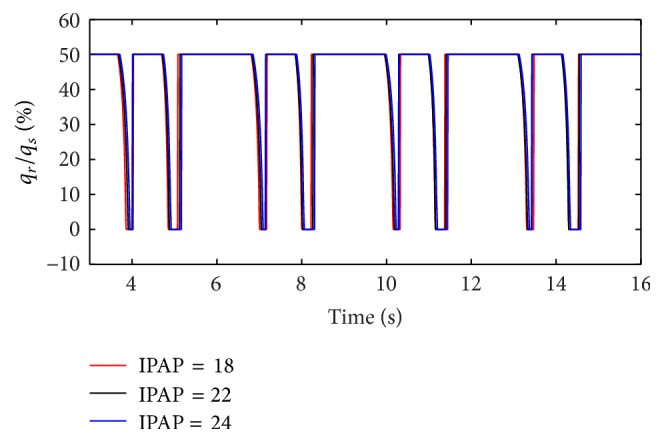
Influence of IPAP on air mass flow when *d* is set to 4.0 mm.

As presented in Figures 9 and 10, increasing IPAP may lead to a distinct rise in the ratio of air mass flow in right lung simulator (*q*_*r*_) to two lung simulators in total (*q*_*s*_) in the inspiration period. As shown in Figure 11, with a rise in IPAP, the ratio of tidal volume of right lung simulator (*V*_Tr⁡_) to the tidal volume of two lung simulators in sum (*V*_Ts_) increases more sharply. When the respiratory compliance (*C*) of right lung simulator is smaller than 10 mL/cmH_2_O, the growth trends of the three curves are almost the same.

(*2) Influence of IPAP under Different Diameters (d) on Dynamic Characteristics*. When the diameter (*d*) of the effective area of the equivalent right throttle is set to 2.4 mm and 4.0 mm, the result of the ratio of air mass flow is shown as in Figures 12 and 13.

**Figure 14:**
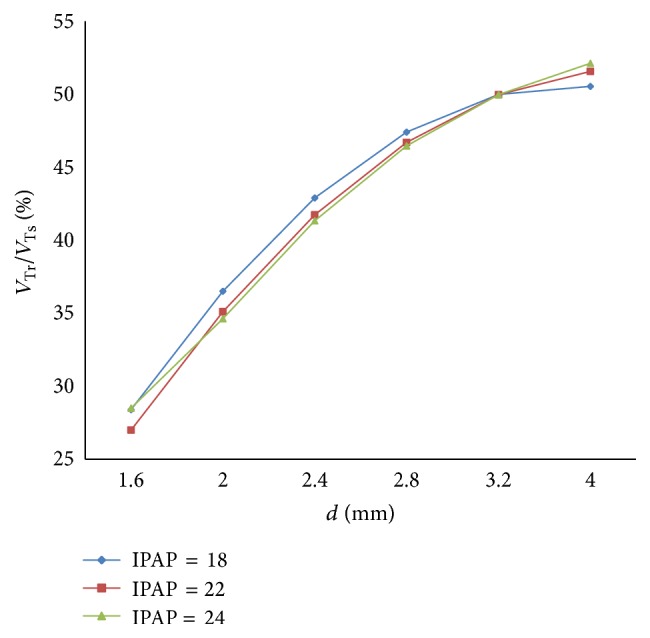
Influence of IPAP on tidal volume of different *d*.

With an increase in the diameter (*d*) of the effective area of the equivalent right throttle, the results of the ratio of tidal volume under different IPAP are shown in Figure 14.

From Figures 12 and 13, it is observed that increasing IPAP can lead to a distinct rise in the ratio of air mass flow in right lung simulator (*q*_*r*_) to two lung simulators in total (*q*_*s*_) in the inspiration period under the condition of different diameter (*d*) of the effective area of the two equivalent throttles. Furthermore, as presented in Figure 14, with IPAP increasing, the curve of the percent of tidal volume becomes steep.

#### 4.2.2. Influence of EPAP on Dynamic Characteristics

**Figure 15:**
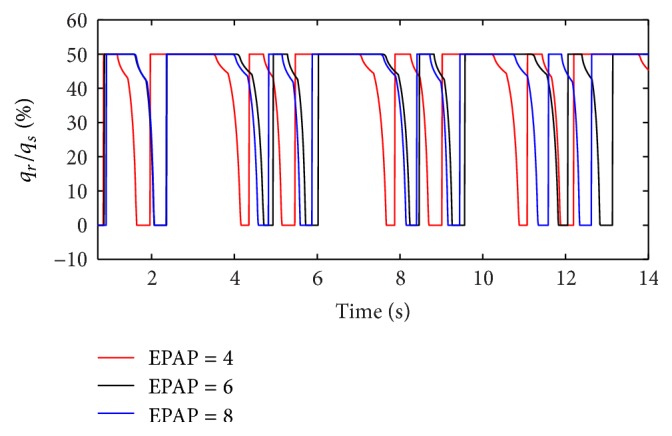
Influence of EPAP on air mass flow when *C* is set to 5 mL/cmH_2_O.

**Figure 16:**
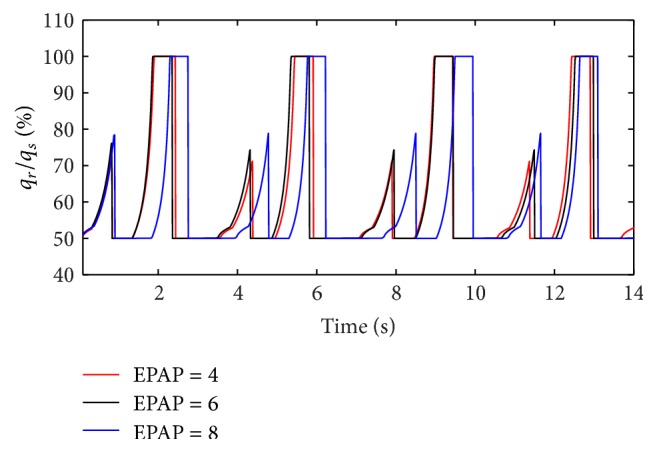
Influence of EPAP on air mass flow when *C* is set to 20 mL/cmH_2_O.

The EPAP is set to 4 cmH_2_O, 6 cmH_2_O, and 8 cmH_2_O. And the simulations are illustrated below.

(*1) Influence of EPAP under Different Respiratory Compliance (C) on Dynamic Characteristics*. When the respiratory compliance (*C*) of right lung simulator is set to 5 mL/cmH_2_O and 20 mL/H_2_O, the effect of EPAP on the ratio of air mass flow is shown as in Figures 15 and 16.

**Figure 17:**
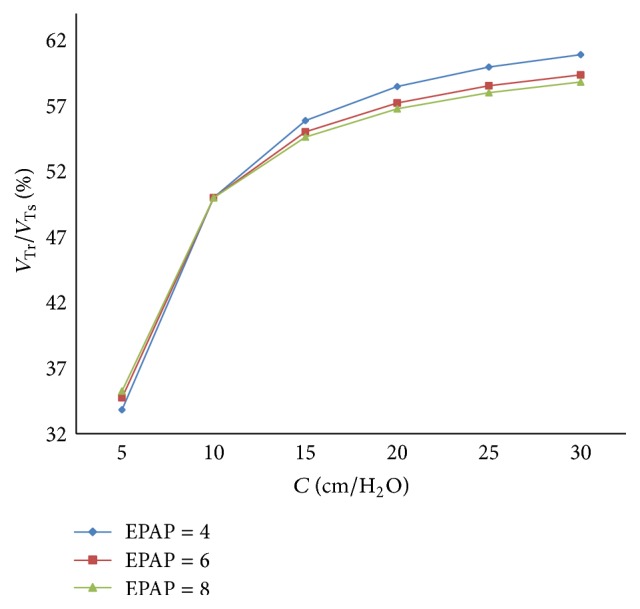
Influence of EPAP on tidal volume of different *C*.

With an increase in the respiratory compliance (*C*) of right lung simulator, the results of the ratio of tidal volume under different EPAP are shown in Figure 17.

**Figure 18:**
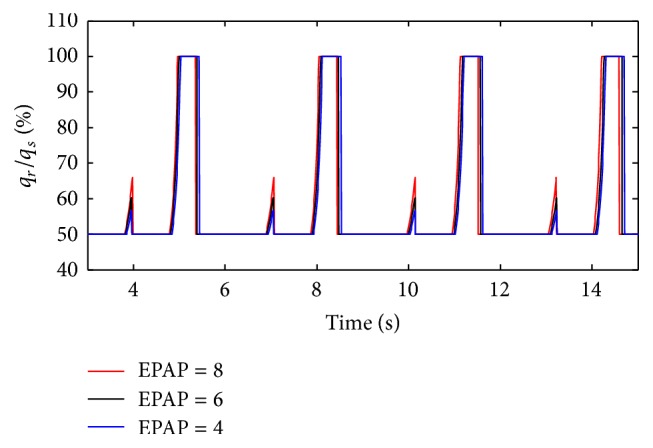
Influence of EPAP on air mass flow when *d* is set to 2.4 mm.

**Figure 19:**
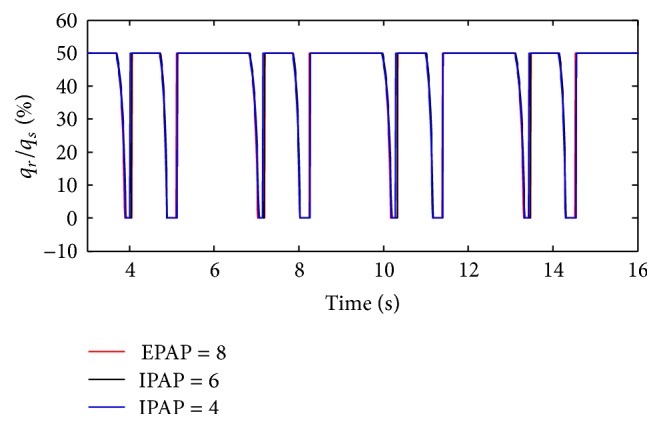
Influence of EPAP on air mass flow when *d* is set to 4.0 mm.

As shown in Figures 15, 16, and 17, increasing EPAP may lead to a distinct rise in the ratio of air mass flow in right lung simulator (*q*_*r*_) to two lung simulators in total (*q*_*s*_) in the inspiration period. When the respiratory compliance (*C*) of right lung simulator is larger than 10 mL/cmH_2_O, the curve of tidal volume ratio rises sharply.

(*2) Influence of EPAP under Different Diameters (d) on Dynamic Characteristics*. When the diameter (*d*) of the effective area of the equivalent right throttle is set to 2.4 mm and 4.0 mm, the effect of EPAP on the ratio of air mass flow is shown as in Figures 18 and 19.

**Figure 20:**
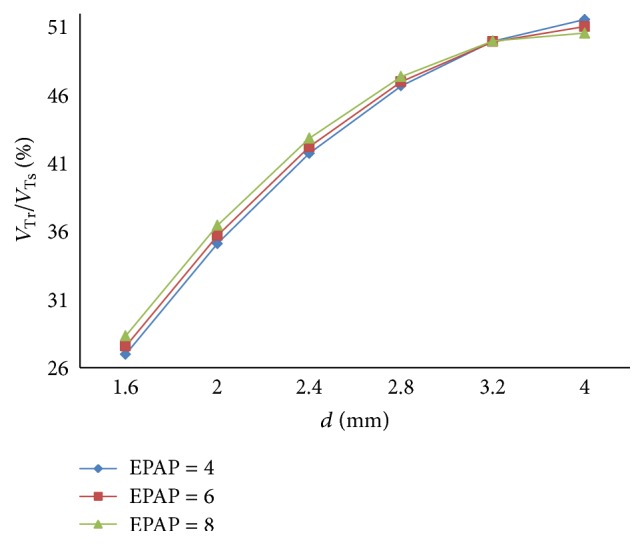
Influence of EPAP on tidal volume of different *d*.

With an increase in the diameter (*d*) of the effective area of the equivalent right throttle *i*, the results of the ratio of tidal volume under different EPAP are shown in Figure 20.

As well as IPAP, the rising EPAP can make a higher percent of air mass flow in the inspiration process. However, to the ratio of tidal volume of right lung simulator (*V*_Tr⁡_) to tidal volume of two lung simulators in total (*V*_Ts_), EPAP make a little difference on it. When the diameter (*d*) of the effective area of the equivalent right throttle is bigger than 3.2 mm, the larger EPAP is, the more slowly the curves increase.

#### 4.2.3. Influence of BPM on Dynamic Characteristics

**Figure 21:**
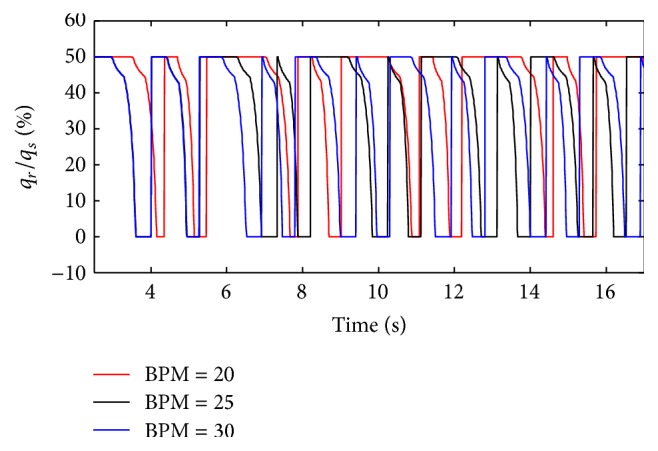
Influence of BPM on air mass flow when *C* is set to 5 mL/cmH_2_O.

**Figure 22:**
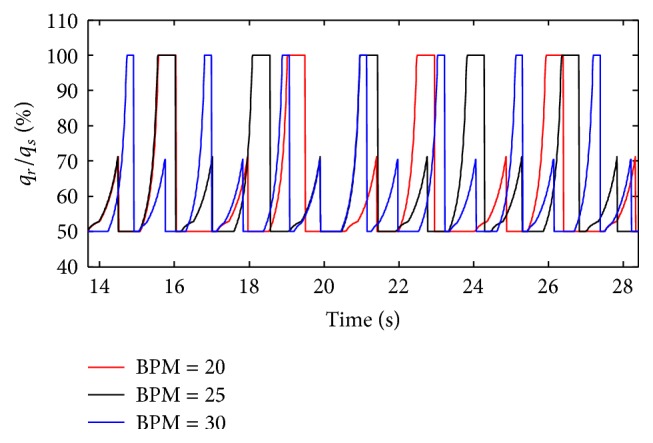
Influence of EPAP on air mass flow when *C* is set to 20 mL/cmH_2_O.

The BPM is set to 20, 25, and 30, while the other parameters of ventilator do not change. The simulation results are shown below.

(*1) Influence of BPM under Different Respiratory Compliance (C) on Dynamic Characteristics*. The influences of BPM on the ratio of air mass flow are shown in Figures 21 and 22, when the respiratory compliance (*C*) of right lung is set to 5 mL/cmH_2_O and 20 mL/cmH_2_O.

**Figure 23:**
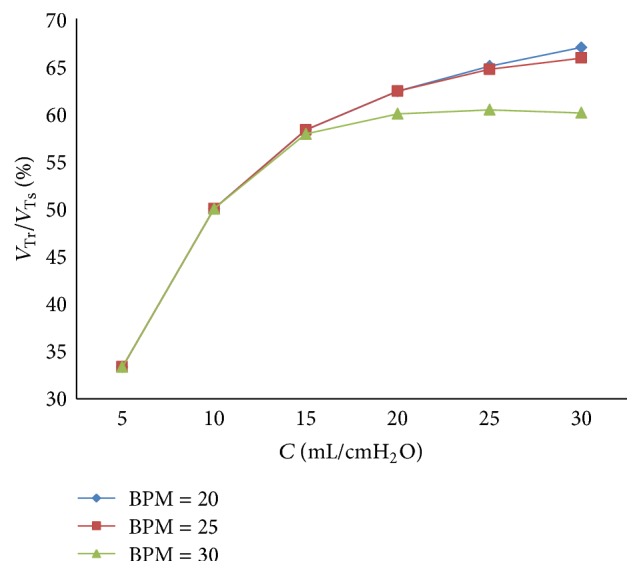
Influence of BPM on tidal volume of different *C*.

With the increase of the respiratory compliance (*C*) of right lung the trend of tidal volume ratio can be gotten in Figure 23 when BPM is 20, 25, and 30.

**Figure 24:**
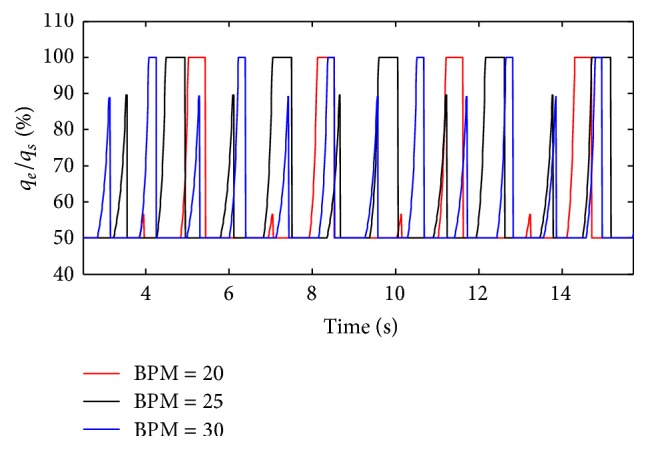
Influence of BPM on air mass flow when *d* is set to 2.4 mm.

**Figure 25:**
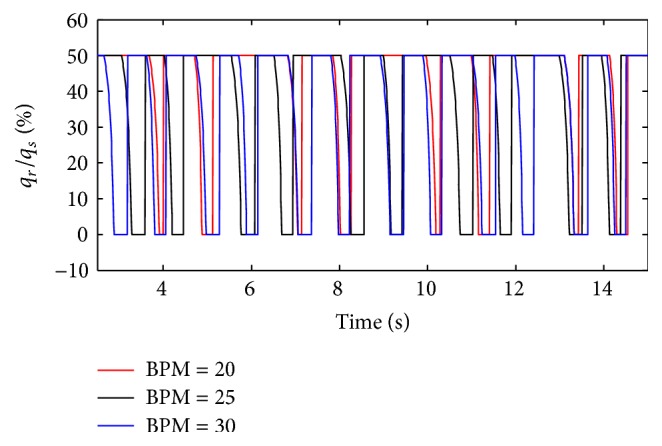
Influence of BPM on air mass flow when *d* is set to 4.0 mm.

As shown in Figures 22 and 23, BPM of ventilator do not make a difference on the value of the ratio of air mass flow; it just affects the cycle of the ventilation system. From Figure 23, when the respiratory compliance (*C*) of right lung simulator is larger than 15 mL/cmH_2_O, the larger BPM is, the more slowly the curves rise.

(*2) Influence of BPM under Different Diameters (d) on Dynamic Characteristics*. The effects of different BPM on the ratio of air mass flow are shown in Figures 24 and 25 when the diameter (*d*) of the effective area of the equivalent right throttle is 2.4 mm and 4.0 mm.

**Figure 26:**
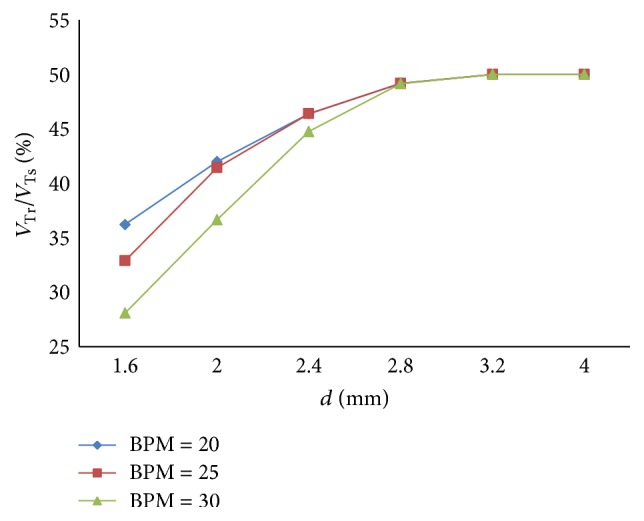
Influence of BPM on tidal volume of different *d*.

With different diameters (*d*), the results of the ratio of tidal volume under different BPM are shown in Figure 26.

As presented in Figures 24 and 25, BPM also just produce an effect on the cycle of the ventilation system, and the effect on other dynamics can be neglected. As shown in Figure 26, with a rise in BPM, the three curves increase obviously. When the diameter (*d*) of the effective area of the equivalent right throttle is smaller than 2.8 mm, the smaller BPM curve has a higher percent of tidal volume. As BPM increases, the growth of the curves becomes rapid.

#### 4.2.4. Influence of Inspiration Time (*T*_*i*_) on Dynamic Characteristics

**Figure 27:**
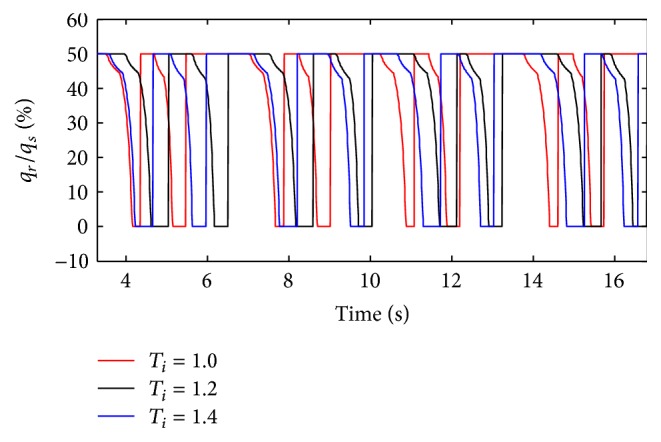
Influence of *T*_*i*_ on air mass flow when *C* is set to 5 mL/cmH_2_O.

**Figure 28:**
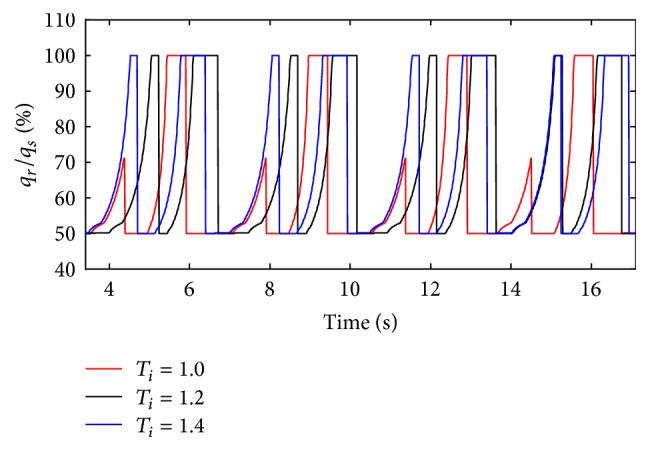
Influence of *T*_*i*_ on air mass flow when *C* is set to 20 mL/cmH_2_O.

The inspiration time (*T*_*i*_) of the ventilator is set to 1 s, 1.2 s, and 1.4 s. The simulation results are shown below.

(*1) Influence of T*_*i*_ *under Different Respiratory Compliance (C) on Dynamic Characteristics*. When the compliance (*C*) is 5 mL/cmH_2_O and 20 mL/cmH_2_O, the influence of different *T*_*i*_ on air mass flow can be seen in Figures 27 and 28. With an increase in the respiratory compliance (*C*) of right lung simulator, the results of the ratio of tidal volume under different *T*_*i*_ are shown in Figure 28.

**Figure 29:**
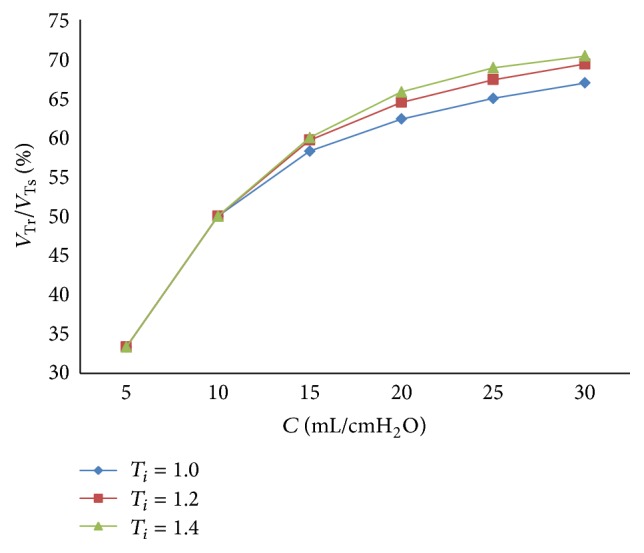
Influence of *T*_*i*_ on tidal volume of different *C*.

**Figure 30:**
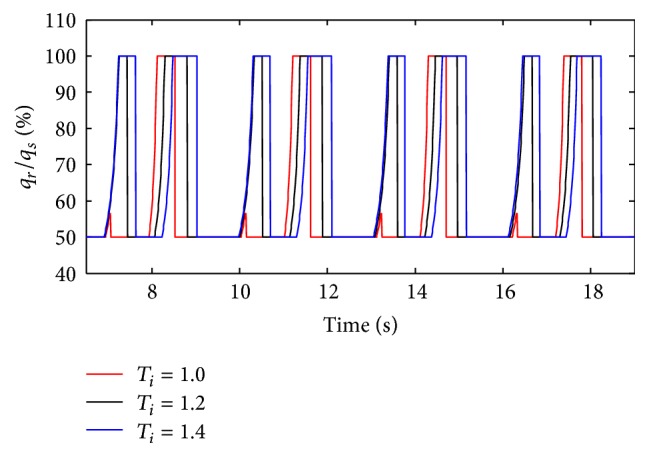
Influence of *T*_*i*_ on air mass flow when *d* is set to 2.4 mm.

**Figure 31:**
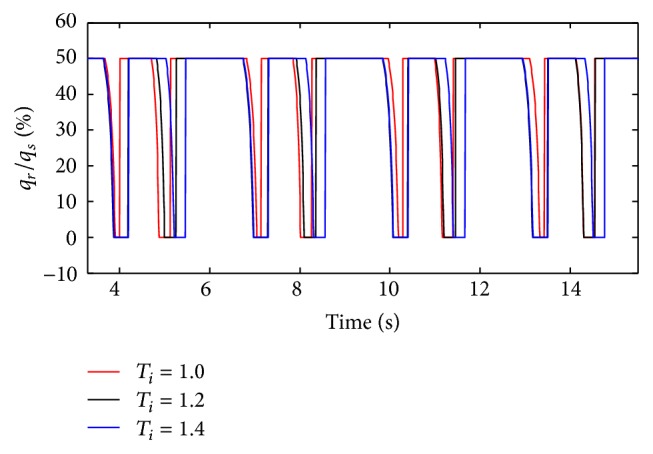
Influence of *T*_*i*_ on air mass flow when *d* is set to 4.0 mm.

**Figure 32:**
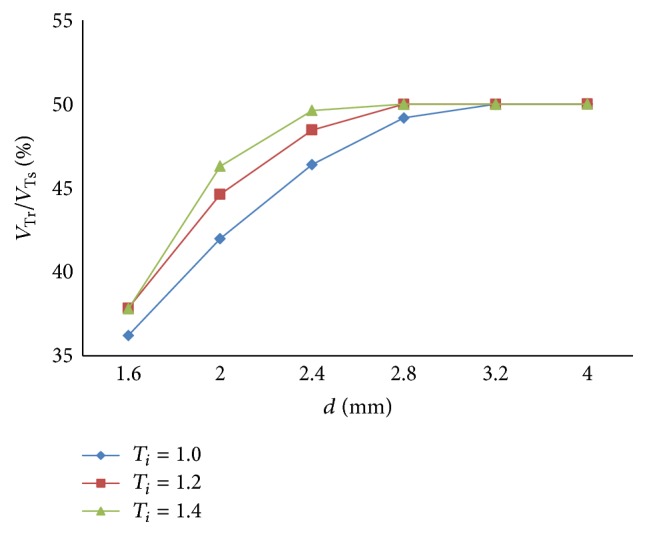
Influence of *T*_*i*_ on tidal volume of different *d*.

As shown in Figures 27 and 28, the inspiration time (*T*_*i*_) of the ventilator only has an effect on the inspiration time of the lung simulator, and its influences on other dynamics are negligible. As illustrated in Figure 29, when the respiratory compliance (*C*) of right lung simulator is bigger than 10 mL/cmH_2_O, the curves increase sharply as a rise in BPM.

(*2) Influence of T*_*i*_ *under Different Diameters (d) on Dynamic Characteristics*. When *T*_*i*_ is 1.0 s, 1.2 s, and 1.4 s, the effects on air mass flow can be given by Figures 30 and 31 under different diameters (*d*). And the influence on tidal volume when diameters (*d*) are set to 1.6 mm to 4 mm can be seen in Figure 32.

When the diameter changes, the inspiration time (*T*_*i*_) of the ventilator just also affects the inspiration time of the lung simulator, and its effects on the other dynamics are negligible. However, to the ratio of tidal volume, the longer the time the inspiration spends, the higher the percent the curve would get.

#### 4.2.5. Influence of the Pressure Rise Time (*T*_*r*_) on Dynamic Characteristics

**Figure 33:**
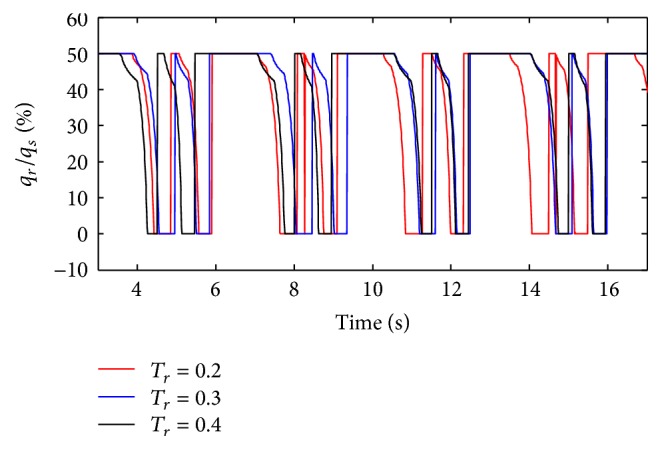
Influence of *T*_*r*_ on air mass flow when *C* is set to 5 mL/cmH_2_O.

**Figure 34:**
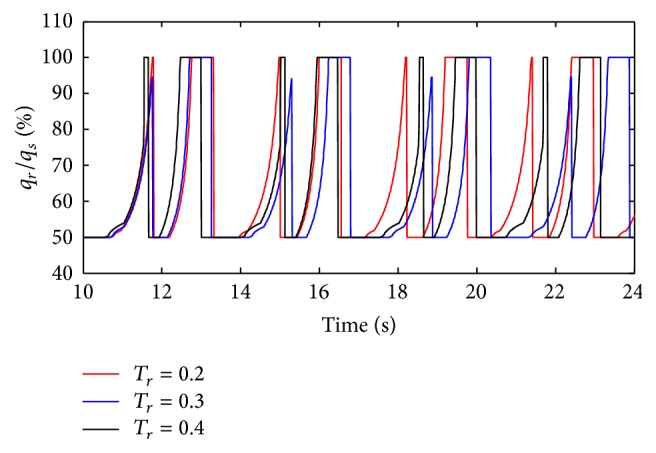
Influence of *T*_*r*_ on air mass flow when *C* is set to 5 mL/cmH_2_O.

**Figure 35:**
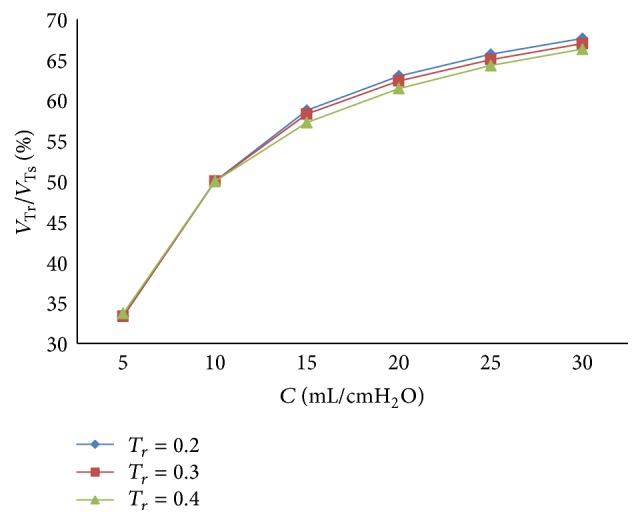
Influence of *T*_*r*_ on tidal volume of different *C*.

The pressure rise time (*T*_*r*_) of the ventilator is set to 0.2 s, 0.3 s, and 0.4 s. The simulation results are shown below.

(*1) Influence of T*_*r*_ *under Different Respiratory Compliance (C) on Dynamic Characteristics. *As shown in Figures 33 and 34, the influence of *T*_*r*_ on air mass flow under different respiratory compliance (*C*) can be seen.  Figure 35 shows the trend of tidal volume with the increase of respiratory compliance (*C*) under three different *T*_*r*_.

**Figure 36:**
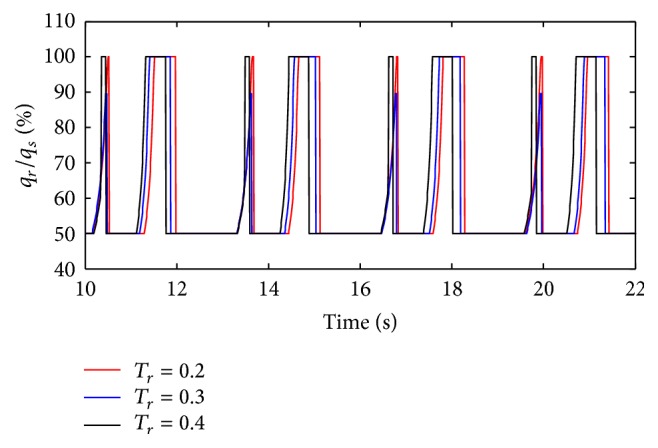
Influence of *T*_*r*_ on air mass flow when *d* is set to 2.4 mm.

**Figure 37:**
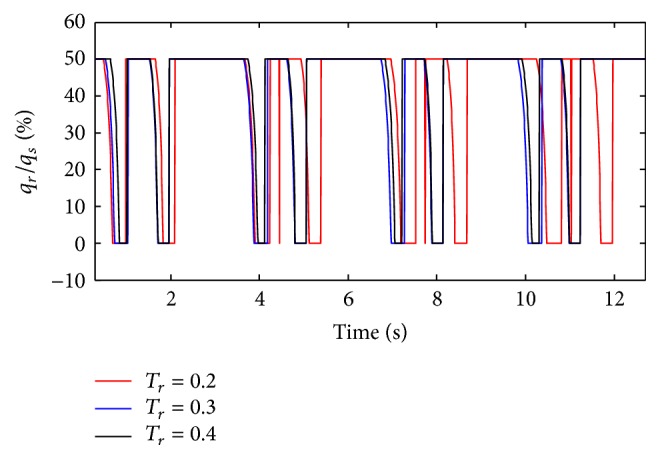
Influence of *T*_*r*_ on air mass flow when *d* is set to 4.0 mm.

**Figure 38:**
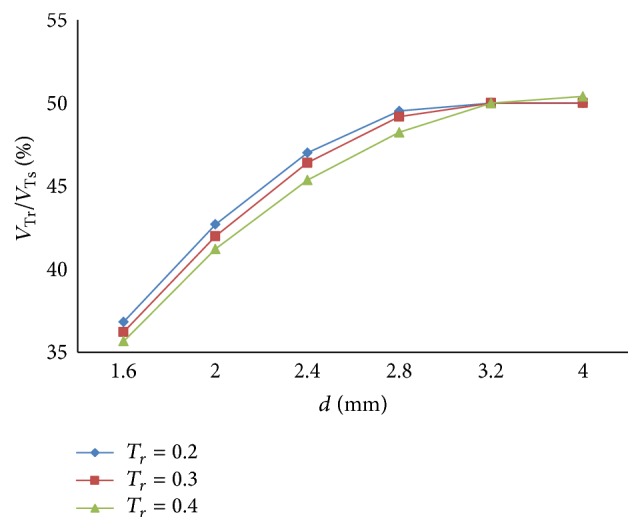
Influence of *T*_*r*_ on tidal volume of different *d*.

From Figures 33 and 34, it is observed that the air flow increases with a decrease in the rise time (*T*_*r*_); the ratio of air mass flow increases with a decrease in the pressure rise time (*T*_*r*_) in the inspiration period. And with an increase in rise time (*T*_*r*_), the time when the ratio of air mass flow stays to 50% becomes longer. From Figure 36, it is observed that the shorter the rise time (*T*_*r*_) spends, the more sharply the curve will increase.

(*1) Influence of T*_*r*_ *under Different Diameters (d) on Dynamic Characteristics*. As shown in Figures 36 and 37, the effects of *T*_*r*_ on air mass flow under different diameters (*d*) can be seen.  Figure 38 shows the trend of tidal volume with the increase of diameters (*d*) when *T*_*r*_ is 0.2 s, 0.3 s, and 0.4 s.

From Figures 36 and 37, it is observed that with an increase in rise time (*T*_*r*_), the time when the ratio of air mass flow stays to 50% becomes longer. As shown in Figure 38, increasing rise time (*T*_*r*_) may lead to a sharp curve of the tidal volume ratio. But the shorter rise time (*T*_*r*_) may produce bigger percent of tidal volume until the diameter (*d*) of the effective area of the equivalent right throttle is smaller than 3.2 mm.

## 5. Discussion

According to the description above, the conclusion can be gotten that the mathematic model of the system is correct. And each of the five key parameters has a regular effect on dynamic characteristics of the mechanical ventilation system with double lungs. As the system is based on two lungs, which increases its applicability and versatility, the dynamic characteristics must be more comprehensive and precise than the other studies based on single lung. The coupling effects of double lungs on dynamic characteristics of the system are more significative to the security and effect on respiratory medicine researches as well.

## 6. Conclusions

In this study, BIPAP ventilation system of double lungs was regarded as a pneumatic system, and then a new mathematical model of the BIPAP ventilation system was derived. To verify the mathematical model of the system, an experimental prototype of BIPAP mechanical ventilation system with double lungs was proposed. Then the influence of different parameters of two lung simulators and ventilator on dynamic characteristics of BIPAP mechanical ventilation system was studied. The conclusions are summed up as follows.

(1) The simulation results are consistent with experimental results. So it verifies the mathematical model of the system which can be used in BIPAP ventilation system of double lungs.
(2) With an increase in the respiratory compliance (*C*) of one lung simulator or the diameter (*d*) of the effective area of the one equivalent throttle, both the ratio of air mass flow and ratio of tidal volume would rise.
(3) When the respiratory compliance (*C*) of one lung simulator is bigger than the other one, or the diameter (*d*) of the effective area of the one equivalent throttle is smaller than the other one, the curves of the ratio of air mass flow and tidal volume would fluctuate from 0% to 50%. On the contrary, the curves may fluctuate from 50% to 100%.
(4) Increasing IPAP and EPAP may lead to a distinct rise in the presence of air mass flow in inspiration. But to the ratio of tidal volume, the increasing IPAP and EPAP may lead to a slow growth.
(5) Influences of BPM, the inspiration time (*T*_*i*_), and the pressure rise time (*T*_*r*_) on the dynamics of the air mass flow ratio are very slight. However, they all have a huge impact on the ratio of tidal volume. The bigger the BPM is, the smaller the ratio of tidal volume will be, as well as the pressure rise time (*T*_*r*_). But increasing inspiration time (*T*_*i*_) can raise both the ratio of air mass flow and the ratio of tidal volume.

The study reveals the dynamic characteristics of mechanical ventilation with double lungs, and it can be referred to in the development of research on BIPAP ventilation treatment and real respiratory diagnostics. The research may promote research on the development of new respiratory clinic treatment.

## Nomenclature

*A*_*e*_: Effective area of throttle (m^2^)
*b*: Critical pressure ratio = 0.528
*C*: Respiratory compliance (L/cmH_2_O)
*d*: Diameter of effective area (m)
*l*: Length (m)
*m*: Mass of air (kg)
*p*: Pressure (pa)
*q*: Air mass flow (kg/s)
*R*: Gas constant = 287 (J/(kg·K))
*R*_*r*_: Respiratory resistance (cmH_2_O/L/s)
*T*: Temperature (K)
*t*: Time (s)
*V*: Volume (m^3^)
*λ*: Friction coefficient
*ρ*: Density (kg/m^3^)
*k*: Specific heat ratio = 1.4.
